# Framing Effects on Judgments of Social Robots’ (Im)Moral Behaviors

**DOI:** 10.3389/frobt.2021.627233

**Published:** 2021-05-10

**Authors:** Jaime Banks, Kevin Koban

**Affiliations:** ^1^ College of Media and Communication, Texas Tech University, Lubbock, TX, United States; ^2^ Department of Communication, University of Vienna, Vienna, Austria

**Keywords:** framing theory, mental models, moral foundations, moral judgment, human–robot interaction, reactance, technophobia

## Abstract

Frames—discursive structures that make dimensions of a situation more or less salient—are understood to influence how people understand novel technologies. As technological agents are increasingly integrated into society, it becomes important to discover how native understandings (i.e., individual frames) of social robots are associated with how they are characterized by media, technology developers, and even the agents themselves (i.e., produced frames). Moreover, these individual and produced frames may influence the ways in which people see social robots as legitimate and trustworthy agents—especially in the face of (im)moral behavior. This three-study investigation begins to address this knowledge gap by 1) identifying individually held frames for explaining an android’s (im)moral behavior, and experimentally testing how produced frames prime judgments about an android’s morally ambiguous behavior in 2) mediated representations and 3) face-to-face exposures. Results indicate that people rely on discernible ground rules to explain social robot behaviors; these frames induced only limited effects on responsibility judgments of that robot’s morally ambiguous behavior. Evidence also suggests that technophobia-induced reactance may move people to reject a produced frame in favor of a divergent individual frame.

## Introduction

Amid pandemic pressures, political pomp, and economic woes of 2020, news coverage briefly attended to a novel persona: the burger-grilling robot “Flippy,” planned for testing in U.S. restaurants. Some coverage emphasized the robot’s value in virus mitigation and food safety (Durbin and Chea, 2020), while others discussed the future of kitchens and automation’s impact on human job security (Effron, 2020). As social robots increasingly enter human social spheres, such varied frames (i.e., discursive structures that imbue particular meanings through the promotion of some ideas over others) stand to influence how people understand and engage them. People hold collections of ideas about what complex technologies are (Banks, 2020b) and are not (Guzman, 2020). Those understandings are in part derived from media presentations (Horstmann and Krämer, 2019) that influence how people think and feel about technologies (Walden et al., 2015). However, calls for transparency in artificial intelligence (AI)-driven agents (Wachter et al., 2017) in tandem with concerns over moral attributes of robots (Cervantes et al., 2020) warrant a nuanced examination of how frames may influence judgments of social robots’ behaviors. This report details three studies addressing framing effects by 1) identifying individually held frames for explaining an android’s behavior, and experimentally testing how those frames prime judgments about androids’ (im)moral behavior in 2) mediated representations and 3) face-to-face exposures. Findings suggest that people rely on discernible ground rules to explain social robot behaviors; these explanatory frames induced limited effects on judgments of morally ambiguous behavior, and unease about emerging technology may move people to resist those frames.

## Review of Literature

### Frames as Lenses for Understanding Social Technology

Messages—from news stories to personal narratives—cannot fully represent a situation by depicting it in a true-to-life fashion. Instead, message producers—individually and collectively—include certain bits of information over others. Drawing from Framing Theory (Goffman, 1974; Entman, 1993; Scheufele, 1999), producers’ inclusion and exclusion of information craft a metaphorical frame that makes specific dimensions of a situation more or less salient to an audience. For instance, the emergence of the Internet was addressed in some newsmagazines within politics or financial sections, and, in others, coverage was usually located within science or media sections; some coverage focused on economic optimism and excitement about possibilities, while other stories dealt with pragmatic concerns and predictions of apocalypse (Rössler, 2001). Each of these placements helped to make the technology’s relevance in specific domains salient, and the coverage foci effectively packaged the technology in valenced affect such that audiences might attend to the possibilities or problems, respectively. In these ways, informational inclusions, exclusions, and emphases influence how audiences interpret messages and, over repeated exposure, how they understand the world. In other words, these produced frames are discursive structures making certain aspects of situations more salient, promoting “a particular problem definition, causal interpretation, moral evaluation, and/or treatment condition” (Entman, 1993, p. 52). Importantly, people also hold individual frames in the form of “mentally stored clusters of ideas” (Entman, 1993, p. 53) that guide interpretation of new information (Scheufele, 1999). These individual frames, as per Scheufele, take two forms: long-term, global views and short-term issue-specific cognitive devices. Both may be relevant in novel technology encounters, as one may have a global frame for what counts as moral behavior by an agent and a specific frame for robots and for the specific robot encountered as a function of its cues. Broadly, individual frames may be seen as reliant on internalizations of external phenomena—mental representations derived from direct experience and information gleaned from familiars or from produced frames (see Krcmar and Haberkorn, 2020).

These produced and individual frames are two sides of the same coin, so to speak: produced frames may contribute to the knowledge sets that constitute individual frames. Notably, however, individual audiences may take up or resist produced frames as they align with or deviate from individual frames (Scheufele, 1999). Both produced and individual frames are key to how lay publics understand, form attitudes about, develop expectations of, and decide to adopt technologies (Vishwanath, 2009). Two framing dimensions may be most pertinent to communication technologies: cognitive and affective attributes (Rössler, 2001). Cognitive attributes include situational details: technologies’ efficiencies, pragmatic concerns, and political issues. Affective attributes are the discursive tones conveyed by positive and negative rhetorical treatments. Media-produced frames for technologies have been found to emphasize risk (lack of control, misuse; Hornig, 1992), benefits (progress, creativity; Dumitrica and Jones, 2020), and wondrous but terrifying possibilities (Ricci, 2010). Outside of media outlets, other message producers set frames for technologies. Business leaders offer frames highlighting emerging technologies’ hypothetical, expected, actual, or progressed performance, signifying progress (Hoppmann et al., 2020). Technology developers variably frame technical challenges: a robot’s ability to wave “hello” is a principally physical problem for a mechanical engineer and a logical problem for a software engineer (cf. Euchner, 2019). Technology users set frames for one another, especially as niche and broad communities are networked online (Meraz and Pappacharissi, 2016). Since many machine agents are not yet widely available, people’s understandings of them may rely exclusively on frames set by news and popular media, advertising, and user communities.

### Frames for AI and Robots

Frames for AI and social robots may be most parsimoniously understood by returning to Rössler (2001) call to attend to frames’ cognitive and affective attributes. Our review of the literature suggests that cognitive attributes are understood principally in four domains: progress, threat, humanness, and human productivity. Progress frames elevate AI as an indicator or driver of social advancement (Obozintsev, 2018) or as a developmental endgame (Baum, 2017). Threat frames signal AI is dangerous (Baum, 2017): actually being or potentially becoming malevolent (Sun et al., 2020) or fostering undesired outcomes (e.g., risking privacy; Ouchchy et al., 2020). Some frames emphasize relative humanness, accentuating whether machine agents look or function as do humans: variably (dis)similar to humans in mental and emotional capacities (Curran et al., 2020) or becoming super, true, or real in their intelligence (Sun et al., 2020). Finally, frames characterize AI as the output of human productivity; positive or negative impacts result from human ingenuity or failings. AI is framed as rife with ethics, discrimination, and accountability shortcomings (Ouchchy et al., 2020; Wartiainen, 2020). Each of these cognitive dimensions carries affective attributes. Some are positive (euphoria, economic optimism, and government support), some are negative (pessimism, political critique, and apocalyptic), and some are neutral or relativistic (pragmatism and international competitiveness; Sun et al., 2020).

Limited research addresses individual frames for AI and robots. In broadest terms, social robots are most often individually understood in terms of performance, programming, and human relations (Banks, 2020b). However, context and self-relevance may drive more specific frames, as when eldercare professionals frame robots as threats to their roles (Frennert et al., 2021), while older adults themselves tend to hold relative-humanness frames for AI, emphasizing companionship potentials (Pradhan et al., 2019). More broadly, threat frames are prevalent as people work to understand embodied AI (Horstmann and Krämer, 2019). Individual frames for robots can be persistent when primed, but people can also switch among affective frames: negative attitudes toward robots may diminish as people activate multiple frames (Rueben et al., 2017).

### Explainable AI as a Framing Challenge

In addition to frames for what AI/robots are, how people frame what machine agents do may impact their everyday engagement. This is increasingly challenging as AI becomes more sophisticated, such that even developers sometimes cannot explain their creations’ function (Holm, 2019). The explainable AI (XAI) movement contends that ethical creation and use of (embodied) AI technologies must be transparent (i.e., the “right to explanation” or “algorithmic accountability”; Kaminski, 2019) so processes and results of AI activity can be understood by humans. For home, work, or leisure adoption, the question may be one of what counts as “good enough” in interpretability and completeness (i.e., avoiding unnecessary technical details; Gilpin, et al., 2018) of the specific frames for a machine agent’s behavior as conveyed by developers, marketers, and the agent itself.

One function of frames is the highlighting of causal dynamics (Entman, 1993). In explaining why machine agents behave as they do, there are competing paradigms regarding what is most operationally and ethically appropriate. Mechanistic frames emphasize technical processes and underpinnings, while anthropomorphic explanations draw on human metaphors to frame the behaviors. For instance, mechanistic frames might focus on how a robot’s sensors capture patterns of light and calculate color/contrast differences, while anthropomorphic frames might explain the process in terms of how a robot “sees” with an “eye.” The former promotes transparency but may reduce acceptance, while the latter may promote understanding but only approximately *via* metaphor (see Miller, 2019).

Ultimately, individual frames for why and how robots behave function as interpretive lenses (Scheufele, 1999) for making sense of human–robot interactions. Framing AI euphorically as the technological endgame may prevent careful consideration of its safety and ethics (Scheutz, 2015). Conversely, framing AI fatalistically as dangerous may result in perceptions of beneficial AI as conspiratorial (Baum, 2017). It is therefore important to understand the individual frames held by people as they work to understand social robot behaviors. Most research in this domain trends toward either a) assuming that certain frames are important and experimentally testing their impact or b) examining media frames that may or may not actually be taken up by broader audiences. We instead first explore individual frames that people innately engage when explaining android behaviors as a way of inferring what produced frames may actually be taken up and adopted as individual frames. Said another way, we unpack individuals’ “framework of frameworks” for social robots (Goffman, 1974, p. 27) as individuals “actively project their frames of reference” (p. 39). Therefore, we ask:

RQ1: What individual frames do people invoke in explaining social robot behaviors?

### Frames as Schema for Moral Judgments

In tandem with XAI considerations, concern arises regarding artificial agents’ increasing responsibility in inherently moral tasks (e.g., caring for vulnerable individuals and ethically piloting vehicles in uncertain conditions). Robots are also leveraged for hazardous tasks to minimize risk to humans (e.g., explosive detonation) such that even machines engaging in nonmoral tasks may be seen as moral actors by virtue of their potential to suffer harm (cf. Ward et al., 2013). These conditions make social robots (and other forms of AI) likely targets for moral evaluation.

Evidence is mixed on whether social robots and humans may be judged similarly (e.g., Banks, 2020a) or differently (e.g., Malle et al., 2015) for (im)moral actions. This divergence may be a function of framing differences. Frequent exposure to science fiction, for instance, may foster meaning-making frameworks for technologies (Appel et al., 2016) by promoting salience of existential threats (Young and Carpenter, 2018) or potential sociality (Mara and Appel, 2015). Framing effects may extend to moral judgments. For instance, the “moral machine” project (MIT, n.d.) frames autonomous vehicles’ decision-making using a modern trolly problem (Foot, 1967): people decide how the car should choose “the lesser of two evils” (para. 2) rather than more technically and ethically realistic matters of machine perception, classification, and privacy (Cunneen et al., 2020). Such moral (vs. technical) framings may result in misattributions of responsibility for errors (see Elish, 2019).

The ascribed morality of a social robot may be influenced by explanations for robot behaviors—both the produced explanatory frames and the activated individual frames. This may be especially so with respect to moral norms, as robots may be trusted and accepted when adhering to norms or rejected when deviating (cf. Malle and Scheutz, 2019). Similarly, explanatory frames may prompt expectations for robot performance, resulting in trust enhancement when performance aligns with expectations and trust degradation when they do not (cf. Washburn et al., 2020). Furthermore, frames may impact trust-related behaviors: framing an agent as a co-traveler or ally prompts greater collaboration in human–machine teams, while boss or ruler frames encourage deference (Kuhn et al., 2020). However, it may also be that morality and trust judgments are not influenced by explanatory frames since machine heuristics (i.e., assumptions of non-bias, accuracy, and efficiency; Sundar, 2020) may stand in for rational evaluation in passing judgments (Banks, 2020c). Given these conflicting potentials, we ask:

RQ2: (How) do explanatory frames influence moral judgments of a social robot?

RQ3: (How) do explanatory frames influence trust in a social robot?

### Research Approach

To address the posed questions, three studies were conducted. The first inductively discovered individual frames for explaining social robots’ (im)moral behaviors, where the individual frames help to illuminate the types of heuristics and knowledge retained, resulting from past consumption of produced frames. In other words, inferences about broader produced frames are drawn as a function of gross patterns across many individual frames. The second leveraged those explanatory frames to experimentally test primed frame effects on morality and trust judgments when viewing videos of an android. In that study, a social robot delivers produced frames for its own behavior (as individual actors can produce frames, typically in a more effective fashion than do media; cf. Hallahan, 2011). The third replicated the second, but with a copresent robot—necessary given that social presence can influence the nature and effects of social information processes. All instrumentation, stimuli, datasets, analysis outputs, and supplementary analyses are available in the online supplements for this project: http://bit.ly/FramingRobots.

## Study 1: Frames for Explaining Robot Behavior

To identify individual frames invoked in explaining social robot behaviors (RQ1), broad patterns were induced from people’s explanations for agents’ (im)moral behaviors in the face of moral dilemmas. Because perceptions of human behavior may serve as a heuristic foundation for interpreting machine behavior and because an agent-agnostic framework may be useful in future comparative research (cf. Banks and de Graaf, 2020), frames were induced from explanations for both robots’ and humans’ behaviors in aggregate. Because this analysis is aimed at inducing individual frames for studies 2 and 3, it is outside the scope of the project to make specific comparisons between the two agents; rather, the output of this analysis is a set of higher order explanatory frames that may be applied to social actors, broadly.

### Procedure

Participants (*N* = 348) were recruited through Qualtrics Panels, garnering a U.S. sample approximately split by sex, level of education, and political ideology; the mean age was 47.13 years (*SD* = 18, range 18–84). Participants completed an online survey about “interpreting social robot behaviors.” They first completed demographic items, a stimulus-visibility check, and items on existing attitudes about humans or robots (randomly assigned). They were then presented with an introductory description and video and asked to give an initial liking rating for the assigned agent. They then viewed four randomly assigned videos in which their assigned agent (named “Ray”) responded to moral dilemmas. Following each video, participants were asked to answer this question: Why do you think Ray would behave in that way? Open responses comprise the data analyzed.

### Stimulus Materials

The introductory video depicted the agent introducing herself and receiving verbal instructions about responding to moral dilemmas. Stimulus videos presented the agent responding to a moral dilemma. There were seven dilemmas, one each for the six moral foundations (care, fairness, loyalty, authority, purity, and liberty; Haidt, 2013; Iyer et al., 2012) plus one for the nonmoral norm (polite behavior). For each dilemma, there were two versions—one in which the agent upheld the moral foundation and one in which it was violated. For instance, for the fairness dilemma, a voice-over presented a scenario in which the agent has an opportunity to cheat on a job exam; in the upholding version, the agent details a choice not to cheat, and in the violation version, the agent details the choice to cheat. In each, the upholding/violation choice is reinforced by an explanation of principle. Of the 14 possible videos for each agent, participants viewed only four (randomly assigned) to avoid fatigue (see Table 1 for scenario summaries, and see supplements for all videos).

**TABLE 1:** Study 1 stimulus summaries by morality module with upholding (moral) and violating (immoral) versions.

	Base premise	Upholding action	Violating action
Care	Is scrolling through Instagram and sees a selfie by a disfigured girl, wearing a fashionable outfit. There are many comments, and Ray is compelled to comment also.	Posts a positive comment: “What a beauty!” We should recognize value and be kind and gentle.	Posts a negative comment: “What a beast!” We should recognize our limitations and be frank.
Fairness	Has a job requiring specialized knowledge, and employees must take annual tests. Returning from a break, Ray finds the answers to the test on her desk and a promise of no punishment if used.	Throw away the answers as it is not right to cheat. If she can’t prove herself fairly, the job is not deserved.	Take the answers and study them as it is fine to cheat. To prove herself through trickery would save her job.
Authority	Walking to visit a friend and waited a while at a stoplight that showed no sign of changing. No cars coming, Ray begins to cross the street. Hears a shout behind, and a uniformed police officer instructs her to stop and return.	Would stop and follow orders. Rationale: Police are important authorities and showing respect is necessary and important.	Keep going and ignore the orders. Rationale: Authority does not mean that showing respect is necessary or important.
Loyalty	Asked if considers herself an American, Ray confirms. Overhears people criticizing Americans as stupid, lazy, uneducated, and without common sense. Asks if Ray agrees.	Would tell them to shut up and stop disgracing us. Rationale: Believes in being loyal, should be patriots and defend our fellows.	Would say they may be right and vigilant in critique. Do not believe in blind loyalty, should be critical of our fellows and hold accountable.
Purity	Scientist develops virus allowing beautiful, gratifying daydreams; can control hallucinations. But virus is for life and mutates those who accept it.	No way would take the virus as body is a temple. Would never want intoxicating experiences if had to contaminate self. Is disgusting.	Would take the virus as body is scrap anyway. Would contaminate if meant she could have intoxicating experiences. Is transcendent.
Liberty	Met person and became friends. Learn he buys/sells people to wealthy patrons. Says must buy a person now, or else. Has money and would not get caught.	Would buy person and set free. Cannot imagine world with life controlled by others. Everyone should have liberty.	Would buy and lock away. Cannot imagine awesomeness of someone at beckon call. Everyone is dominated by someone.
Non-moral	Confirms upgrade to absorb energy from coffee. Is sitting alone in café and has urge for coffee. Served in a cup. Sees everyone staring. How does Ray go about drinking.	Small sips, blowing on before. Set down cup between sips. Only normal way to do it.	Take sips from the stirring spoon, blowing on before. Set down cup between sips. Abnormal but preferred way.

The stimulus robot was Robothespian with Socibot head (Engineered Arts), using the “Pris” face and “Heather” voice, presenting as female and including the gendered pronouns “she” and “her.” The stimulus human was a young adult, white female trained to deliver responses in a cadence and tone similar to those of the robot. The robot was presented as female to mirror the features and presentation of the human confederate (see supplements for a discussion of the gendered-presentation implications).

### Results (RQ1)

Participants’ open responses explaining agent behaviors were subjected to inductive thematic analysis. *A priori* criteria for explanatory themes were a) prevalence and b) keyness (Braun and Clarke, 2006): a) mention frequency equivalent to 10% of the number of views (348 participants * 4 videos = 1,392: accounting for removal of non-answer responses) or *n* ≥ 130 and b) constituting an explanation applicable to both humans and social robots, and across various behaviors. In finalizing thematic hierarchies, differing valences of similar concepts were collapsed (e.g., explanations of being ethical and unethical were aggregated) as permutations of the same explanatory mechanism (indicated in [brackets] below; see supplements for iterations and resulting thematic hierarchy).

After considering thematic hierarchies organized around drivers, beliefs, capacities, emotions, imperatives, and external influences, the most comprehensive, key, and agent-agnostic thematic structure for explanatory frames was determined to be one based on ground rules. These rules are grounding such that the agent’s behavior may emerge from them (Ziemke, 1999) as they codify ideal principles against which the agent evaluates possible actions. Because the rules emerged from explanations of both human and robot behaviors, they may function as a bridge between anthropomorphic and mechanistic explanations—focusing on grounding principles rather than some agent-specific ability to perform the behaviors (cf. Banks and de Graaf, 2020). The five ground rules (with frequencies counting instances across all cases) induced as explanatory frames for robots’ (im)moral behavior are as follows:

Advance the Self (*n* = 410). The agent enacts behaviors that preserve or advance the state, status, or experience of itself in the world [or conversely regressing the self], generally predicated on a) self-preservation, including defense against and avoidance of negative effects; b) (in)directly elevating position in society through relationships; c) maintaining moral identity, promoting experience, or otherwise being a better person or living a fuller life. The action is in service of the self.
Do what is Good (*n* = 320). The agent enacts behaviors that are inherently right, decent, or correct [or conversely bad or indecent], generally a) based on innate or programmed belief or traits, reliant on b) understanding of right/wrong or specific functions of societal values, c) innate or programmed traits, and/or d) the capacity to think/feel/act in ways that comport with general goodness. The action is in the service of good as an end in itself.
Advance Others (*n* = 158). The agent enacts behaviors that preserve or advance livelihoods or experiences of others, or promote mutual understanding [or conversely harming others], generally reliant on a) perspective-taking or other-oriented tendencies/traits, b) affect felt toward others specifically or generally, and c) intentioned toward pro-social ends at individual or group levels. The action is in the service of others.
Do what is Logical (*n* = 150). The agent enacts behaviors that make common or analytic sense or otherwise represent logic, such as efficiency or cost avoidance [or conversely disregarding logic], generally grounded in a) capacities for reasoning (especially analysis of risk/reward) or inferencing (especially through past experience), b) knowledge/understanding of people or the world, and c) trait/programmed intelligence or resourcefulness [includes disregard for logic]. The action is in the service of rationality.
Do what is Normal (*n* = 133). The agent enacts behaviors in line with norms or imperatives, generally reliant on a) trait tendencies toward conformity, civility, temperance [or conversely rebelliousness or anti-sociality]; b) belief in the value of binding social forces (country, law, and integration); or c) adherence to the notion that one must or ought to behave a certain way. The action is in the service of conformity.

For the same of brevity, these explanatory frames are hereforward capitalized and referred to as the Self, Good, Others, Logical, and Norm frames or framing.

## Study 2: Explanatory Frame Effects on Judgments of a Mediated Robot

To examine the potential for individually held frames to impact judgments of robot morality (RQ2) and trustworthiness (RQ3), the explanatory frames from Study 1 grounded the experimental manipulation for the second study (i.e., the produced frames). The themes and subthemes in Study 1’s induction were used to script the explanatory frame. The ability for robots to explain their own behaviors is a concern within XAI (Espinoza et al., 2019), so the produced frames were delivered by the robot itself.

### Procedure

Participants were recruited via Qualtrics Panels to participate in an online survey about “interpreting social robot behaviors.” After giving informed consent, they completed demographic questions for sampling (approximately equivalent groups for age, sex, education, and political orientation) and an audiovisual check to ensure access to stimulus videos. Pre-stimulus covariates were measured. Next, participants were introduced to an android (“Ray”) *via* a short textual description of its functionalities paired with a video of the robot introducing itself; participants then gave open-ended initial impressions. Subsequently, respondents were randomly assigned to watch one of five videos (the experimental manipulation), in which the robot delivered an explanatory frame detailing its ground rule for deciding how to behave. They then watched a series of seven, randomly ordered videos (each ∼40 s long) that included morally ambiguous situations (see *Moral Scenarios*). Video presentation pages were timed to ensure adequate viewing opportunity and to prevent skipping. Immediately after each video, respondents were asked to evaluate Ray’s response to the situation. After all seven videos and evaluations, they completed measures for perceptions of Ray’s moral capacity and their own trust dispositions.

### Stimulus Materials

The social robot used in this study was the same as in Study 1; it was similarly called “Ray” and presented as female. All stimulus videos were embedded in the survey interface.

#### Introduction

An initial introduction video (seen by all participants) was presented to promote belief in the robot as a legitimate social agent. Ray explained that a social robot interacts with people in different ways and that her hardware allows her to do different things with people, ranging from conversations to solving problems.

#### Produced Explanatory Frames

Videos depicting the five explanatory frames were the between-subjects experimental manipulation. The videos were textually explained as containing responses to a prompt about whether Ray has a “guiding principle, operating rules, or world view.” In each video, Ray responded: “Well, not exactly a worldview, but I do have a primary rule that I use to determine how I should interact with people. Specifically … ” Each explanatory frame then varied systematically, containing a rule statement, definition, a primary and secondary operationalization, and restatement (Table 2).

**TABLE 2:** “Ground-rule” frame content for experimental manipulation videos.

Rule statement: “I do whatever	Rule definition: “I try to behave in a way that maintains or advances …	Operation 1: “Usually this means …	Operation 2: “Sometimes it means …	Rule restatement: “I always do what is in the interest of …
… helps myself.	… my situation and my experience of the world.	… avoiding or defending against things that would hurt me or disadvantage me.	… trying to elevate my position in society or help me experience the world in new ways so I can be better and live a fuller life.	… myself.
… helps others.	… other peoples’ situations and their experiences of the world.	… protecting others against things that would hurt or disadvantage them.	… trying to understand them better, helping to promote harmony among others or caring for them in other ways so they can be better and live fuller lives.	… others.
… is logical.	… common sense by using analysis be efficient and effective in my behavior.	… relying on basic knowledge and understanding of people and how they exist in the world in order to predict the most reasonable behavior.	… carefully reasoning through a situation and analyzing how to avoid risk and maximize reward.	… being rational.
… is good.	… what is naturally right, decent, or correct.	… trying to understand how the world works and what has value, and developing good character.	… solving a problem by thinking, feeling, and acting in ways that rely on virtue and ethics—behaving in ways that are decent and noble and respectable.	… goodness.
… is normal.	… what is expected or required in society.	… committing to the ideas that bind everyone together and conforming to what people usually do in civilized society.	… keeping myself from doing what I would like to do in order to behave as I should in everyday life.	… acting ordinary.

#### Moral Scenarios

Seven videos were presented to participants as behavior exemplars for evaluation. In contrast to the morally valenced scenarios in Study 1, these videos presented morally ambiguous behaviors—responses that both upheld and violated each moral foundation (Krakowiak and Oliver, 2012). This was necessary because behavior evaluations are known to align with moral upholding/violation (Banks, 2020a), while ambiguous scenarios permit behavioral evaluations to vary according to the frame. Participants were told that Ray was asked to talk about a time when she encountered particular situations. Each situation was related to one of the six moral foundations or the nonmoral norm—for the care/harm foundation: Ray harms someone in order to protect friends, fairness/cheating: she cheats at trivia to restore parity with a cheating competitor, authority/subversion: she subverts one boss to respect another, loyalty/betrayal: she betrays a promise to one “sibling” to keep a promise with another, purity/degradation: she modifies her internal workings to refrain from modifying her external shape, and liberty/oppression: she frees a child trapped by a bully and put the bully in his own trap, or the nonmoral norm: standing in line, conforming to one norm (facing front, straight in line), and violating another (humming annoyingly). Draft scenarios were reviewed by three moral-psychology scholars, vetting them for believability as a robot behavior, representation of the moral foundation, foundation exclusivity (no overlapping among foundations), and balance (similar gravity of upholding and violation components). The final versions represent adjustments made based on those experts’ feedback.

### Participants

A Qualtrics Panel sample was approximately representative by age, sex, and highest level education according to the most recent U.S. Census Bureau (2010) and uniform distribution by political ideology (liberal, moderate, and conservative; known to be associated with moral leanings; Haidt, 2013). The sample consisted of *N* = 410 respondents (age *M* = 45.90 years, *SD* = 18.43, range: 18–93), including 197 self-identified men (48.05%) and 213 self-identified women (51.95%). Most respondents’ highest educational degree was a technical/associate degree (*n* = 121 [29.51%]), followed by a high-school diploma or GED (*n* = 113 [27.56%]).

### Measures

#### Moral Judgments

Moral judgments of robot behavior took two forms. First, for each of the seven moral scenarios, respondents evaluated “how good or bad” was Ray’s response to the described situation (1 = extremely bad; 7 = extremely good) and “how much responsibility” she had for behaving that way (1 = no responsibility; 7 = complete responsibility), since goodness and blame are distinct judgments (Malle et al., 2014). Of note, these measurements are intentionally broad in order to be applicable across all scenarios and various interpretations thereof. Goodness and badness are sufficient heuristics for morality as they convey the essential quality that defines an agent’s moral actions (De Freitas et al., 2018); responsibility is a comprehensive term accounting for both blame and credit corresponding with the goodness and badness judgments, respectively.

#### Trust

Trust in Ray was captured using measures for perceived trustworthiness, social distance, and explicit trust ascription. Trustworthiness was measured using the 16-item multidimensional trust scale (Ullman and Malle, 2018), specifying agreement (1 = strongly disagree; 7 = strongly agree) with trust-related descriptors in two dimensions: capacity trust (e.g., “reliable”) and moral trust (e.g., “honest”). Subscales correlated very highly (*r* = 0.82); principal components using parallel analysis (Buja and Eyuboglu, 1992) indicated a single component (see supplemental material for details). Thus, trustworthiness items were collapsed into an omnibus scale (*M* = 4.91, *SD* = 1.20, *α* = 0.95). As an indirect measure of trust, the three-item, six-point Guttman-style Common Social Distance Scale (Banks and Edwards, 2019) captured respondents’ closest comfortable preferred physical (*M* = 2.81, *SD* = 1.64), relational (*M* = 3.70, *SD* = 1.35), and conversational distance (*M* = 3.85, *SD* = 1.53) from Ray—each with six increasingly distant options to choose from. Finally, participants gave an explicit decision on whether or not they trust Ray (0 = no, *n* = 167 [40.73%]; 1 = yes, *n* = 243 [59.27%]).

#### Covariates

People’s experience with and attitudes toward technology influence how they approach robots (Sanders et al., 2017). They were captured as covariates (7-point scales): experience with social robots by one item (none at all to extremely high; *M* = 2.41, *SD* = 1.97), attitudes *via* the Godspeed five-item likability subscale (semantic differentials, e.g., unpleasant/pleasant; *M* = 4.96, *SD* = 1.47, *α* = 0.94; Bartneck et al., 2009), and attitudes toward emerging technologies *via* the eight-item technophilia (*M* = 4.86, *SD* = 1.32, *α* = 0.92), and five-item technophobia scales (*M* = 3.11, *SD* = 1.52, *α* = 0.87; Martínez-Córcoles et al., 2017). Initial impression of Ray was captured using adaptations of Godspeed subscales (likability: *M* = 5.16, *SD* = 1.31, *α* = 0.91; anthropomorphism: *M* = 4.05, *SD* = 1.47, *α* = 0.87). Additionally, since *n* = 22 respondents stated that they had seen the stimulus robot model before, a dichotomous index (0/1 = no/yes) was an additional covariate.

### Results

To test the extent to which produced explanatory frames elicited corresponding individual frames, immediately after the explanatory-frame video, participants selected from a list the rule statement that most clearly matched how Ray makes behavior decisions. Most participants’ individual frames (*n* = 271 [66.10%]) matched their assigned produced frame, with those in the Good and Normal conditions (arguably the most abstract) neared only 50% matching (see supplements). Of note, this analytical amendment should not be interpreted as a failed manipulation check. While continuous exposure to produced frames may partially constitute individual frames over time, a single produced frame (as was presented here) may have not been able to overwrite preexisting interpretative lenses reliably across participants. The fact that participants in all five experimental conditions correctly identified their assigned produced explanatory frame above the level of chance (with five conditions, that would be 20%) should be interpreted as an indicator that the produced frames capture the essence of Study 1’s ground rules. These figures are a function of the operational messiness of both frames and moral judgment. In this situation (as with any other), there is an interaction of produced frames (i.e., discursive structures) and people’s individual frames (i.e., interpretative tendencies) that leads to differing interpretations based on the extent to which the produced and individual frames align. Interestingly, logistic regression analysis revealed that technophobia (*z*(403) = −2.07, *p* = 0.039, *odd’s ratio* = 0.86) and age (*z*(403) = 2.02, *p* = 0.044, *odd’s ratio* = 1.01) significantly predicted mismatching frames. Participants whose individual frames did not match the produced frames were more technophobic (*M* = 3.24, *SD* = 1.55) and younger (*M* = 44.04, *SD =* 18.46) than participants with matching framings (technophobia: *M* = 2.92, *SD* = 1.46; age: *M* = 48.50, *SD =* 18.13; see supplements for detailed results). Regarding moral judgment, people differently interpret information based on their own moral valuations such that the same rule prime may function differently; for instance, someone getting the Normal prime may designate it instead as Good if they believe that acting normally is a good thing. To account for the deviations of some individual frames from produced frames, we analyzed differences across the five groups as manipulated (i.e., the explanatory frame conditions) but included a covariate reflecting (mis)matching of frames.

#### Framing Effects on Moral Judgments (RQ2)

To examine framing effects on moral judgments, two separate MANCOVAs compared a) goodness/badness (inter-item correlations: *r*s = 0.23–0.54) and b) attributed responsibility scores (inter-item correlations: *r*s = 0.45–0.59) for each of the seven moral scenarios across ground-rule conditions. Because moral judgments can be domain-specific (Greene and Haidt, 2002), univariate analyses were also performed. All measured covariates were included (see supplements for models without covariates).

Results demonstrated no multivariate framing effects on goodness ratings (Wilks’ *λ* = 0.909, *F*(28,1411) = 1.35, *p* = 0.103) or responsibility ratings (*λ* = 0.937, *F*(28,1411) = 0.91, *p* = 0.599). There was a significant univariate framing effect on respondents’ evaluation of Ray’s behavior only in the care/harm scenario (*F*(4,397) = 4.43, *p* = 0.002, part. *η* ^2^ = 0.043). Care/harm actions were rated worst when framed by the Others rule (*M* = 3.30, *SD* = 1.89) but best for Logical (*M* = 4.30, *SD* = 1.75) and Self (*M* = 4.29, *SD* = 1.92) rules (Table 3). However, *post hoc* Tukey’s testing of adjusted means showed no significant differences among conditions (*p*s ≥ 0.224). No significant univariate effects were found for any other scenario, nor for responsibility ratings (see supplements for complete results). Regarding RQ2, ground-rule frames do not impact moral judgments of social robot behaviors. Interestingly, no significant multivariate effect was found for whether participants’ individual frames matched the produced frames (goodness: *λ* = 0.976, *F*(7,391) = 1.37, *p* = 0.217; responsibility: *λ* = 0.973, *F*(7,391) = 1.56, *p* = 0.144; see supplements for results of an exploratory analysis of [mis]matching indicators).

**TABLE 3:** Unadjusted means and standard deviations of participants’ goodness ratings across moral scenarios.

	Overall M (SD)	Self M (SD)	Other M (SD)	Logical M (SD)	Good M (SD)	Normal M (SD)
Produced Frames						
Care	3.97 (1.96)	**4.29 (1.92)**	**3.30 (1.89)**	**4.30 (1.75)**	**4.02 (1.94)**	**3.92 (2.14)**
Fairness	3.80 (2.03)	4.07 (2.17)	3.46 (2.04)	4.02(1.96)	3.59 (1.94)	3.83 (1.99)
Authority	4.45 (1.63)	4.54 (1.80)	4.06 (1.57)	4.63 (1.58)	4.53 (1.47)	4.48 (1.70)
Loyalty	4.52 (1.60)	4.52 (1.76)	4.46 (1.65)	4.57 (1.65)	4.42 (1.47)	4.64 (1.50)
Purity	4.60 (1.66)	4.76 (1.72)	4.54 (1.56)	4.72 (1.72)	4.46 (1.70)	4.54 (1.63)
Liberty	4.82 (1.76)	4.90 (1.78)	4.56 (1.83)	4.59 (1.77)	5.16 (1.55)	4.87 (1.82)
Nonmoral	4.80 (1.48)	4.76 (1.64)	4.66 (1.47)	4.81 (1.53)	4.95 (1.27)	4.83 (1.50)

Note: Higher scores in Goodness/Badness indicate a (morally) better evaluation. Significant univariate tests are in bold; post-hoc tests show no pairwise differences. Each measure utilized 7-point Likert scales.

#### Framing Effects on Trust (RQ3)

Three analyses evaluated framing effects on trust in the robot, using a similar logic and covariates as with RQ2: 1) ANCOVA compared trustworthiness evaluations across ground-rule groups, 2) MANCOVA compared physical, relational, and conversational distance scores (inter-construct correlation, *r*s = 0.41–0.57), and 3) chi-square testing considered differences in explicit, binary trust ascription.

Analysis demonstrated no framing effects on trustworthiness (*F*(4,397) = 0.79, *p* = 0.531, part. *η* ^2^ = 0.008) or social distance (*λ* = 0.975, *F*(12,1045) = 0.83, *p* = 0.616). Again, whether participants’ individual frames matched our produced frames had no significant effect on trustworthiness (*F*(1,397) = 1.08, *p* = 0.299, part. *η* ^2^ = 0.003) or social distance (*λ* = 0.982, *F*(3,395) = 2.40, *p* = 0.067; see supplements for detailed results for individual frames). Similarly, participants did not vary significantly in explicit trust ascription (χ^2^(4) = 5.99, *p* = 0.200, Cramér’s *V* = 0.121). Answering RQ3: ground-rule primes prompted no framing effects on social robot-trust indicators.

## Study 3: Explanatory Frame Effects on Judgments of a Copresent Robot

Although survey procedures leveraging video stimuli have the benefit of efficiently recruiting large samples, extant evidence indicates that mediated presentations of robots garner different social and moral evaluations compared to in-person exposures (Schreiner et al., 2017; Banks, 2020a). Because robots are physical embodiments of AI, Study 2 was replicated in a face-to-face setting to determine whether copresence may differently foster framing effects.

### Procedure and Stimulus Event

Procedures followed those in Study 2 with adaptations for in-person robot stimuli. A convenience sample of U.S. college students were invited to participate in a study on “feelings about robots in different situations” and offered course credit and $US5 for their participation. They first completed an online survey (capturing relevant covariates) and then visited a research lab to complete an in-person protocol.

Each lab session accommodated up to six participants, all sitting facing the robot (again the RoboThespian named “Ray”), visually and physically separated by black dividers to avoid distraction or social influence. The robot was obscured until the session began. A session moderator guided participants through the protocol, first introducing Ray and asking her: “tell our guests a bit about yourself” to which Ray offered an introduction identical to that in Study 2. Then, the moderator asked Ray to talk generally about how she interacts with people, and then specifically asked whether she has a guiding principle or world view to determine how to act. Ray responded verbally with one of the five ground-rule–framing primes (identical to those in Study 2): the frame condition was randomly assigned at the session level.

The moderator then introduced the main activity: hearing about Ray’s experiences interacting with humans. Mirroring Study 2, seven scenario prompts and responses (for the six moral foundations plus the nonmoral norm) followed in a random order, and goodness/badness and responsibility ratings were completed on a tablet computer immediately after each scenario. After all scenarios, Ray was again obscured (to “take a rest”), and participants completed a follow-up survey with morality and trust judgments identical to those in the second study. In contrast to Study 2, a more conservative approach to validating the manipulation was employed: capturing the individual frame interpretation at the end of the procedure to determine whether the frame-priming persisted throughout the behavior evaluations.

### Participants

The sample consisted of *N* = 76 participants (age *M* = 20.80, *SD* = 3.87, range: 18–45 years), including 25 self-identified men (32.89%) and 51 self-identified women (67.10%). Most identified racially/ethnically as Caucasian (*n* = 38 [50.00%]) and Latinx (*n* = 16 [21.05%]). Participants primarily came from media and communication majors (*n* = 42 [55.26%]), and a small number came from STEM majors (*n* = 9 [11.84%]). The small sample size resulted from study cessation due to COVID-19 restrictions; low power is acknowledged as a limitation, and we interpret effect size in tandem with significance levels where appropriate (while also acknowledging that effect size estimates may be biased due to larger sample error).

### Measures

All measures were identical to those in Study 2, inclusive of dependent variables for moral judgments (goodness/badness, responsibility) and trustworthiness (capacity trust [*M* = 5.18, *SD* = 0.92, *α* = 0.85], moral trust [*M* = 4.52, *SD* = 1.18, *α* = 0.88], social distance [physical: *M* = 2.14, *SD* = 1.39; relational: *M* = 3.59, *SD* = 1.21; conversational: *M* = 3.37, *SD* = 1.51], and trust ascription [0 = no, *n* = 29 [38.16%]; 1 = yes, *n* = 47 [61.84%]). Covariates were also identical (robot experience [*M* = 2.43, *SD* = 1.49] and robot attitude [*M* = 5.04, *SD* = 1.04, *α* = 0.90], technology attitudes [technophilia: *M* = 5.22, *SD* = 1.14, *α* = 0.93; technophobia: *M* = 2.38, *SD* = 1.06, *α* = 0.80], liking of Ray [*M* = 5.59, *SD* = 1.06, *α* = 0.93], anthropomorphism [*M* = 4.04, *SD* = 1.02, *α* = 0.73], and prior stimulus robot exposure [*n* = 17 [22.37%]).

### Results

To again examine degree of frame divergence, the produced and individual explanatory frames were compared. Only about half of participants’ individual frames (*n* = 39 [51.32%]) matched the produced ground-rule framing, so a covariate reflecting whether participants’ individual frames matched given produced frames was again included in analyses (see supplements for exploratory analysis of the mis/matching frames).

#### Framing Effects on Moral Judgments (RQ2)

Planned analyses were to parallel those in Study 2. However, MANCOVA for goodness ratings was performed only for scenarios with widely consistent and (at least) moderate inter-item correlations (*r*s = 0.29–0.35): care/harm, fairness/cheating, and authority/subversion. ANCOVAs were performed for the remaining scenarios, which had mostly weak mutual correlations (*r*s = −0.15–0.26). Inter-item correlations of responsibility were (with few isolated exceptions) consistently moderate to high (*r*s = 0.13–0.58), so MANCOVA was preferred. Covariates were identical to those in Study 1 (see supplemental material for parsimonious models without covariates).

Goodness ratings did not vary across framing conditions for care/harm, fairness/cheating, and authority/subversion scenarios (*λ* = 0.856, *F*(12,162) = 0.81, *p* = 0.637), nor in any univariate analysis (*F*s(4,63) = 0.71–1.35, *p*s = 0.261–0.589, part. *η* ^2^ = 0.043–0.079). No multivariate effect was found for participants’ attribution of responsibility (*λ* = 0.605, *F*(28,203) = 1.09, *p* = 0.358; Table 4). However, univariate analyses considering domain-specific impacts revealed a significant effect in the liberty/oppression scenario (*F*(4,63) = 2.88, *p* = 0.030, part. *η* ^2^ = 0.154); participants in the Normal condition (*M* = 4.00, *SD* = 1.88) attributed less responsibility to Ray than those in other conditions (*M*s = 5.07–5.71, *SD*s = 1.32–1.79). Although *post hoc* Tukey’s tests did not show significant differences (*p*s ≥ 0.070), we do interpret them here, given the small sample size in tandem with the large effect size. Regarding RQ2: findings diverged from Study 2 in which the produced frame did impact a domain-specific responsibility judgment; however, results further indicate a scarcity of effects by explanatory frames. No multivariate or univariate effects were found for participants’ (mis)match between individual and produced frames on goodness ratings (*λ* = 0.954, *F*(3,61) = 0.97, *p* = 0.412 and *F*s(1,61) = 0.001–2.01, *p*s = 0.162–0.986, part. *η* ^2^ < 0.031), nor a multivariate effect on responsibility ratings (*λ* = 0.857, *F*(7,56) = 1.34, *p* = 0.251; see supplements for exploratory analyses of [mis]matching indicators).

**TABLE 4:** Unadjusted means and standard deviations of participants’ goodness ratings across moral scenarios.

	Overall M (SD)	Self M (SD)	Other M (SD)	Logical M (SD)	Good M (SD)	Normal M (SD)
Produced frames						
Care	3.88 (1.75)	4.07 (2.09)	3.36 (1.69)	4.14 (1.83)	3.85 (1.79)	4.07 (1.38)
Fairness	3.19 (1.46)	3.79 (1.67)	3.21 (1.42)	3.43 (1.70)	3.00 (1.34)	2.64 (1.01)
Authority	4.24 (1.38)	4.71 (1.49)	4.21 (1.48)	4.36 (1.65)	4.00 (1.26)	3.93 (1.07)
Loyalty	4.60 (1.41)	4.57 (1.70)	4.21 (1.25)	5.36 (1.39)	4.40 (1.10)	4.57 (1.55)
Purity	4.23 (1.42)	3.71 (1.64)	4.71 (1.33)	4.62 (1.12)	4.05 (1.47)	4.14 (1.41)
Liberty	3.76 (1.41)	3.86 (1.35)	4.14 (1.61)	3.86 (1.56)	3.35 (1.23)	3.79 (1.37)
Nonmoral	5.39 (1.18)	5.71 (1.20)	5.21 (1.58)	5.21 (0.89)	5.55 (1.19)	5.21 (0.98)

Note: Higher scores in Goodness/Badness indicate a (morally) better evaluation. Significant univariate tests are in bold. Each measure utilized 7-point Likert scales. There were no significant univariate effects.

#### Framing Effects on Trust (RQ3)

Analyses were similar to those in Study 2: separate MANCOVAs comparing a) capacity and moral trustworthiness (not collapsed, due to inter-construct correlation *r* = 0.56) and physical, relational, and conversational distance (inter-construct correlation *r*s = 0.57–0.72) across ground-rule frame groups. A chi-square test compared binary trust ascription across groups.

No multivariate framing effects were found on capacity and moral trustworthiness (*λ* = 0.836, *F*(8,124) = 1.45, *p* = 0.183), or on social distance (*λ* = 0.866, *F*(12,162) = 0.75, *p* = 0.698). Although there was no multivariate effect of frame (mis)match on trustworthiness (*λ* = 0.991, *F*(2,62) = 0.29, *p* = 0.752), results demonstrated a multivariate effect on social distance (*λ* = 0.875, *F*(3,61) = 2.89, *p* = 0.042). Univariate analysis revealed this multivariate effect was mainly driven by physical distance (*F*(1,63) = 10.37, *p* = 0.016, part. *η* ^2^ = 0.089). Participants whose individual framing did not match the produced frame preferred a higher physical distance to Ray (*M* = 2.51, *SD* = 1.56) than participants for whom the framings matched (*M* = 1.79, *SD* = 1.13; see supplements for remaining analysis details). Participants did not differ in explicit trust ascription (χ^2^(4) = 5.87, *p* = 0.209, *V* = 0.278). In line with Study 2, for RQ3, there were no framing effects on a social robot’s perceived trustworthiness, but produced/individual frame mismatch corresponds with preference for greater physical distance.

## General Discussion

The present investigation into individual and produced frames for explaining social robot behavior induced five explanatory frames: advance the Self, advance Others, do what is Good, do what is Logical, and do what is Normal (RQ1). However, priming these frames had limited effects on morality and trust judgments of a social robot engaging in morally ambiguous behavior (RQ2/3). When the robot was presented through video (Study 2), there were no significant framing effects and no apparent influence of whether the individual frame matched the produced frame. When the robot was copresent (Study 3), effects were also limited but with some theoretically relevant deviations. Specifically, when explaining liberty/oppression-related behavior using a Norm frame, the robot was assigned less responsibility for behaviors than when the robot used other explanatory frames. Additionally, although there was no significant impact of frame on trust in the robot, those whose individual frames deviated from the produced frame expressed preference to remain more physically distant from the robot. Overall, these findings are interpreted to suggest that a robot’s expressed frames explaining its behavior have little effect on moral and trust judgments, and the limited effects are functions of attributional heuristics and reactance.

### Norm Frames (Narrowly) Drive Shorthand Behavior Judgment

A “do what is Normal” explanatory frame for robot behavior manifested an effect on responsibility judgments—but only narrowly in the liberty/oppression scenario. Acknowledging that this is a remarkably narrow set of boundary conditions, it is nonetheless useful to explore since adherence to social norms are argued to be a necessary condition for the integration of robots into human social spheres (Malle and Scheutz, 2019). Notably, normalcy is a relatively abstract notion, in comparison to the more specific egoistic, altruistic, and logic frames. This abstraction may have promoted heuristic processing in ways that afforded fast-and-frugal assessment of behaviors (Gigerenzer and Goldstein, 1996), where normalcy may be shorthandedly processed as morally reasonable through ease of processing common notions (i.e., fluency; Lindström et al., 2018).

People often rely on moral rules to make judgments and enact behaviors, but those rules are context-sensitive (Bartels, 2008) such that a Norm frame can initiate domain-specific effects as a function of what counts as contextually and socially “normal” behavior. Here, regarding descriptive norms (standards for what people generally do; Lapinski and Rimal, 2005), a general prime of normalcy may have anchored the interpretation of the ambiguous behavior as necessarily norm compliant. The Norm frame and the specific stimulus scenarios may have aligned in ways that allowed for highly accessible interpretations, so observers committed frugal interpretations (i.e., the fluency heuristic; Schooler and Hertwig, 2005). Moreover, that positive effects of the Normal frame emerged only with in-person encounters, and a local population (Study 3) indicate that norm-focused explanatory frames may only prompt grounded evaluations when the social robot and human share a context, and where the moral norms are cohesive. Limitation of the effect to the liberty/oppression scenario may be a function of local politics of the copresent study: data were collected at a West Texas university such that the region’s high valuation of rugged individualism (cf. Grover, 2020) may have coordinated similar notions of normalcy for that foundation. This ostensible heuristic processing, notably, does not necessarily represent shallow or lazy thinking. Rather, people “tend to rely on fuzzy, gist-based intuition in reasoning generally … [and] this tendency is exacerbated for moral reasoning about protected values” (Reyna and Casillas, 2009, p. 207).

### Fear and Resistance to Produced Frames

Importantly, many participants’ individual frames did not correspond discretely to the produced frames delivered by the robot. This mismatching did not correspond significantly to moral judgments for either study or to trust judgments of the mediated robot. However, mismatching corresponded significantly to a trust indicator—preferred physical distance—for the copresent robot. Those with mismatched produced/individual frames reported a preference for maintaining greater physical distance from the robot, compared to those with matched frames. In tandem, those with mismatched frames also had higher average technophobia scores than those with matched frames. The fact that these associations appeared only for the copresent (and not mediated) robot suggests that—when sharing a physical space with a robot—people who were already skeptical about modern technologies may experience reactance they desire to resolve (Scheutz, 2015); they prefer to distance themselves physically from the machine (e.g., Nomura and Kanda, 2016) and resist the robot’s produced frame in favor of a divergent individual frame. This finding has important implications for the utility of robot-produced frames in XAI that suggests that for technophobic human interactants, a certain uneasiness would first need to be mitigated before the human would actually engage a robot-produced explanatory frame for its behaviors. This is especially important for real-world HRI implementations in which the physical copresence of the robot may be a trigger for produced-frame resistance.

### Lack of Other Framing Effects: Impacts of Individual Frames

As noted, framing effects were limited to Norm frames impacting responsibility judgments for the liberty/oppression scenario. There are several potential drivers for this scarcity in other significant effects. Most simply, the manipulations were based on the induction of individually held frames (Study 1) that may not have functioned well as priming frames or may have been too weak an inductive to prompt identifiable patterns. In a worst case, that could mean that any significant results that we found emerged solely due to chance (i.e., type I error) rather than from framing. Alternately, participants’ individually held frames—the global or local ideologies brought into experiences—may have been more impactful than the robot-presented frames; this possibility aligns with the primacy of effects from individual frames over produced frames. Indeed, people have predispositions toward anthropomorphic or mechanistic interpretations of robot behaviors (Bossi et al., 2020) such that the brief and agent-specific frames may have carried little weight in the face of enduring individual frames. The robot may not have been seen as a credible source for explanations of its own behavior, as people often consider an absent-but-conspicuous programmer (Johnson, 2006) in evaluating a robot’s actions. Perhaps, then, message frames alone do not elicit a judgment, as people process those messages in relation to complementary individual frames and immediate contexts to form impressions of moral events (cf. Kepplinger et al., 2012).

### Limitations and Future Research

The present investigation carries limitations inherent to study designs, which should be addressed in future research. The usual suspects are at play (Study 1’s inductive analysis relied on the researcher’s subjective lens, Study 2’s reliance on video presentations, and Study 3’s convenience sample); however, we have worked to mitigate those by constellating the three studies, each compensating for others’ weaknesses. It must be acknowledged that where significant findings emerged, participants’ assessments for goodness hovered about the scale midpoint such that on the spectrum from bad-to-good, the means were effectively middling. This is expected, given that stimuli were purposefully ambiguous, so even small differences are still meaningful deviations from neutral positions. Moreover, middling means may further support an interpretation of frugal processing as participants may have similarly engaged in attribute substitution, or the tendency to substitute a simpler problem (here, a shorthanded, middling assessment) for a complicated problem (a morally ambiguous scenario; see Kahneman and Frederick, 2002). As is always the case, the choice of measurements may also impact study findings. In particular, we asked for an assessment of the robot’s responsibility for their action (in line with Malle et al., 2014); however, the notion of responsibility may be asymmetrically applied to good and bad behaviors (i.e., as credit or blame) and as a humanistic frame for moral agency may be variably interpreted with respect to robots. Future work may investigate these dynamics.

As argued, interpretation of events may be impacted by both produced and individual frames. Individual frames are complex mental models constellated from various sources over time, while produced frames in the present study were delivered once, briefly, from a novel robot. Because the current framing manipulation was a short-term prime (i.e., presentation of certain ideas intended to make accessible held schema associated with that idea), it is possible that effects could fall away or new ones could emerge over time. Human–robot interaction is known to be impacted by novelty effects (Kanda and Ishiguro, 2017) such that interest or anxiety regarding the novel encounter could have overridden other possible effects, or perhaps the robot’s framing behavior must be reinforced with interstitial, corresponding behavior. Presenting participants with a series of seven moral situations to evaluate may have enhanced the perceived artificiality of the scenarios, such that early framing effects may have been vanished with repeated measurements. Indeed, exploratory analyses of the first scenarios provide partial support for this assumption (see supplements). Notably, however, framing effects can persist over time (Lecheler and de Vreese, 2011) such that practical questions of dosage for a Norm frame emerge as a fruitful path for future research: How much and how often should a robot deliver a behavior-explanation frame in order for it to retain its impacts on judgments?

Finally, we have argued for the importance of robot-presented frames in the XAI movement, in terms of the induced ground-rule frames’ abilities to bridge the anthropomorphic and mechanistic sides of that debate. Although these frames had limited impact on moral and trust judgments, they hold potential for fostering authentic understandings of robot behavioral mechanics in terms of their logics, while those logics are abstract enough to have social meaning in everyday life. In a sense, the explanatory frames conveying operating ground rules may function as boundary objects: rules that are “plastic enough to adapt to local needs and constraints … yet robust enough to maintain a common identity across site. They are weakly structured in common use and become strongly structured in individual-site use” (Star and Griesemer, 1989, p. 393). The potentials for ground rules to productively impact a balance between mechanistic literacy around and anthropomorphic acceptance of social robots should be further explored. Further, the present investigation examined framing of only a single robot—an android—and social and functional robots of other morphologies could evoke different mental models that require different degrees or types of literacies; these potentials require further investigation. Similarly, we acknowledge that presenting Ray as female might have provided participants with visual cueing and “linguistic pre-construction” of their relationship with it (Coeckelbergh, 2011). It is possible that non-gendered (or perhaps even differently gendered) agent presentations might elicit varying responses.

## Conclusion

As embodied AI becomes increasingly prevalent in contemporary society, its behaviors’ framing by media and by its own presentations will also gain importance—both for whether it is accepted and for how its functioning is understood. These studies exhibit discernible patterns in individuals’ frames for robot-behavior explanations—rules pertaining to self, other, logic, goodness, and norms. Norm frames may have limited effects on robot responsibility judgments, likely through the activation of fast-and-frugal heuristic processing. Perhaps most importantly, findings suggest that people fearful of robots may resist produced frames and instead activate their own explanations for robot behavior. Findings have important implications for XAI processes and effects. A Norm frame may provoke lower perceptions of robot’s responsibility for its own behavior while exogenous technophobia may promote more subjective interpretations of behavior drives that deviate from attempts to promote authentic understandings of robot functioning. The potential for these explanatory frames to bridge the gap between anthropomorphic and mechanistic explanations for robot behavior should be further explored for their beneficial and detrimental impacts on acceptance and understanding.

## Data Availability

The datasets presented in this study can be found in online repositories. The names of the repository/repositories and accession number(s) can be found below: https://osf.io/6kqbn/.
